# Increase in Reported Coccidioidomycosis — United States, 1998–2011

**Published:** 2013-03-29

**Authors:** Clarisse A. Tsang, Farzaneh Tabnak, Duc J. Vugia, Kaitlin Benedict, Tom Chiller, Benjamin J. Park

**Affiliations:** Arizona Dept of Health Svcs; California Dept of Public Health; Div of Foodborne, Waterborne, and Environmental Diseases, CDC

Coccidioidomycosis, also known as valley fever, is an infection caused by inhalation of *Coccidioides* spp. spores. This soil-dwelling fungus is endemic to arid regions of Mexico, Central and South America, and the southwestern United States (1). Symptomatic patients typically experience a self-limited influenza-like illness, but some develop severe or chronic pulmonary disease, and less than 1% of patients experience disseminated disease (1). Coccidioidomycosis can be costly and debilitating, with nearly 75% of patients missing work or school because of their illness, and more than 40% requiring hospitalization (2). Previous publications have reported state-specific increases in coccidioidomycosis in Arizona and California during 1998–2001 and 2000–2007, respectively (3,4). To characterize long-term national trends, CDC analyzed data from the National Notifiable Diseases Surveillance System (NNDSS) for the period 1998–2011. This report describes the results of that analysis, which indicated that the incidence of reported coccidioidomycosis increased substantially during this period, from 5.3 per 100,000 population in the endemic area (Arizona, California, Nevada, New Mexico, and Utah) in 1998 to 42.6 per 100,000 in 2011. Health-care providers should be aware of this increasingly common infection when treating persons with influenza-like illness or pneumonia who live in or have traveled to endemic areas.

In collaboration with the Council of State and Territorial Epidemiologists (CSTE), CDC compiles data on selected diseases through NNDSS. Data are reported to CDC from various state and territorial surveillance systems and reporting mechanisms. Coccidioidomycosis has been nationally notifiable since 1995; however, it was not nationally notifiable in 2010. Although the CSTE case definition includes both laboratory and clinical criteria, Arizona uses a laboratory-only case definition because of its large number of cases and the high predictive value of a positive laboratory result (2); since 2008, the laboratory component of the CSTE definition has included cases with a single positive serologic test. California uses the CSTE case definition, requiring both laboratory and clinical evidence of infection, but some counties with large numbers of cases use a laboratory-only definition.

State and regional annual incidence rates were calculated by dividing the number of cases by U.S. Census Bureau population estimates for each year. Crude, sex-specific, age-specific, and age-adjusted incidence rates (aIR) were calculated for Arizona, California, and other endemic states where coccidioidomycosis is reportable (Nevada, New Mexico, and Utah, combined). Rates were age adjusted using the 2000 U.S. standard population. Negative binomial regression was performed to assess statistical significance of incidence trends during 1998–2011. This model adjusts for changes in population size and age and sex distribution over time.

During 1998–2011, a total of 111,717 coccidioidomycosis cases were reported to CDC from 28 states and the District of Columbia: 66% from Arizona, 31% from California, 1% from other endemic states, and <1% from nonendemic states. In Arizona, California, Nevada, New Mexico, and Utah combined, the number of cases increased from 2,265 in 1998 (aIR: 5.3 per 100,000 population) to 8,806 in 2006 (18.0 per 100,000); a decrease occurred in 2007 and 2008 before an increase in 2009 (12,868 cases; 25.3 per 100,000), which continued into 2010 and 2011 (42.6 per 100,000) (Table 1).

Incidence in endemic states increased among all age groups during 1998–2011 (Figure). During this period, incidence typically was highest among the 40–59 year age group in California but was consistently highest among persons aged ≥60 years in Arizona and other endemic states. Incidence during 2011 was 381.1 per 100,000 among persons aged 60–79 years and 385.2 per 100,000 among persons aged ≥80 years in Arizona (Table 2).

During 1999–2008, most (56%) Arizona cases occurred among males, but beginning in 2009, a higher proportion (55%) of cases occurred among females. Incidence in 2011 in Arizona was substantially higher among females (286.9 per 100,000) than males (215.7 per 100,000). In contrast, only 35% of California cases occurred among females during 1998–2011, and 2011 incidence among California males (20.5 per 100,000) was more than double that among females (9.7 per 100,000).

The increase in the number of Arizona cases, from 1,474 in 1998 to 16,467 in 2011, was statistically significant by negative binomial regression (aIR: 30.5 per 100,000 in 1998; 247.7 per 100,000 in 2011, p<0.001). Adjusting for changes in population demographics, this corresponds to an increase in coccidioidomycosis incidence of approximately 16% each year during the study period. The number of California cases increased from 719 in 1998 (aIR: 2.1 per 100,000) to 5,697 in 2011 (aIR: 14.9 per 100,000) (average annual increase of 13%, p<0.001). The number of cases reported in Nevada, New Mexico, and Utah combined increased from 72 in 1998 (aIR: 1.4 per 100,000) to 237 in 2011 (aIR: 3.1 per 100,000) (p<0.001). Cases reported in nonendemic states increased from six in 1998 to 240 in 2011.

## Editorial Note

This report describes statistically significant increases in the incidence rate of reported coccidioidomycosis in endemic states during 1998–2011 after adjusting for changes in population size and in age and sex distribution. Although the number of cases decreased in Arizona during 2007–2008 and in California during 2007–2009, incidence dramatically increased in 2010 and 2011. In 2011, coccidioidomycosis was the second most commonly reported nationally notifiable condition in Arizona and the fourth most commonly reported in California (5).

The reasons for the increases described in this report are unclear. *Coccidioides* exists in the soil and is sensitive to environmental changes; factors such as drought, rainfall, and temperature might have resulted in increased spore dispersal, and disruption of soil by human activity, such as construction, also might be a contributing factor.

Changes in surveillance methodology might have resulted in artifactual increases. California transitioned to a laboratory-based reporting system during 2010, which facilitated reporting and might account for the increase in reported cases in 2011. However, some highly endemic counties, such as Kern County, already had been using laboratory-based systems, so this cannot fully explain the recent increase. The observed increase in Arizona might be partially attributable to a 2009 change by a major commercial laboratory to conform its reporting practices to the 2008 CSTE case definition, whereby positive enzyme immunoassay (EIA) results were reported as cases without confirmation by immunodiffusion. One commercially available EIA test (Meridian Bioscience) commonly used to diagnose coccidioidomycosis has been described to have false-positive results in some instances (6), but the contribution of this phenomenon, if any, to the overall increase in cases is unknown.

Improved awareness of coccidioidomycosis might have resulted in increased diagnostic testing (and thus reporting) in endemic and nonendemic states. *Coccidioides* has been found to be the etiologic agent in an estimated 15%–29% of community-acquired pneumonias in highly endemic areas (7). However, a 2006 study demonstrated that only a small proportion (2%–13%) of patients with compatible illness in an endemic area were tested for coccidioidomycosis (7), suggesting that the disease is greatly underreported. Further study is needed to understand if testing practices have changed. Despite the increase in reported cases, overall U.S. coccidioidomycosis mortality rates have remained fairly stable at approximately 0.6 per 1 million person-years during 1990–2008 (8).

What is already known on this topic?

Coccidioidomycosis is an infection that results from inhalation of *Coccidioides* spp. fungal spores. It is endemic in the southwestern United States, with the highest number of cases occurring in Arizona and California, and constitutes a substantial public health burden in these areas, particularly among older persons.

What is added by this report?

Reported coccidioidomycosis cases have increased dramatically in recent years. The age-adjusted incidence was 5.3 cases per 100,000 population in the endemic area in 1998 and 42.6 per 100,000 in 2011. Among persons aged 60–79 years in the endemic area, incidence was 69.1 cases per 100,000 in 2011.

What are the implications for public health practice?

The number of reported cases of coccidioidomycosis is increasing. Health-care providers should be alert for this infection among persons with influenza-like illnesses who live in or have traveled to endemic areas. Further research on strategies to reduce the morbidity of coccidioidomycosis is needed.

The findings in this report are subject to at least four limitations. First, NNDSS data might underrepresent the actual burden of disease because coccidioidomycosis is not reportable in every state, even in known endemic areas such as Texas, and because state reporting of cases to CDC is voluntary. In particular, the number of cases reported in 2010 might underestimate the actual number of infections because coccidioidomycosis was not notifiable in 2010 (but became notifiable again in 2011). Second, minor discrepancies between the findings in this report and those presented in *MMWR’*s annual *Summary of Notifiable Diseases* reports likely exist because the summary does not include cases from states where the disease was not reportable. Third, minor discrepancies might exist between this report and state-specific reports because of delays in case reporting. Finally, because nearly 70% of cases were missing race/ethnicity data, incidence rates by race and ethnicity were not calculated. This is an important consideration because high rates among Asians and blacks have been documented previously, and black race has been shown to be an independent risk factor for disseminated coccidioidomycosis (9).

Further investigation is needed to determine how much of the observed increase in coccidioidomycosis incidence is artifactual. Nevertheless, health-care providers should be alert for coccidioidomycosis among patients of all ages who live in or have traveled to endemic areas. Persons in endemic areas should consider trying to reduce exposure to dusty air, which might contain *Coccidioides* spp. spores. However, because there are currently no proven preventive measures for coccidioidomycosis, additional research into strategies that reduce the incidence or morbidity of this infection is warranted. Specifically, the role of antifungal treatment for primary pulmonary disease remains controversial and deserves further exploration (10), although treatment is recommended in certain patient groups, particularly those at high risk for severe disease (1). Because the symptoms of coccidioidomycosis mimic those of other community-acquired respiratory illnesses, patients often experience delays in testing and diagnosis and receive unnecessary antibiotics; however, patients who know about coccidioidomycosis are more likely to request testing and receive a diagnosis sooner than those who are not familiar with the disease (2). Therefore, promoting increased community and health-care provider awareness of this infection continues to be an important role for public health officials.

## Figures and Tables

**FIGURE:**
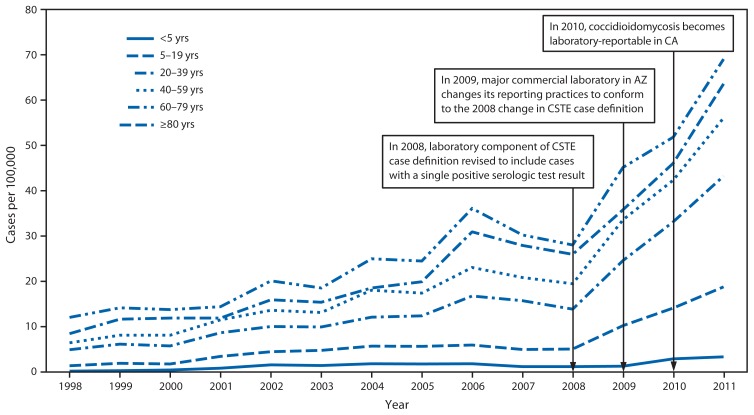
Coccidioidomycosis incidence per 100,000 population, by age group — Arizona, California, Nevada, New Mexico, and Utah, 1998–2011 **Abbreviations:** CSTE = Council of State and Territorial Epidemiologists; AZ = Arizona; CA = California.

**TABLE 1:** Number and age-adjusted incidence per 100,000 population of coccidioidomycosis cases, by region — Arizona, California, Nevada, New Mexico, and Utah, 1998–2011

Year	Arizona		California		Nevada, New Mexico, and Utah		Total endemic area*	
			
	No.	Incidence	No.	Incidence	No.	Incidence	No.	Incidence
1998	1,474	30.5	719	2.1	72	1.4	**2,265**	**5.3**
1999	1,812	37.6	939	2.8	55	0.9	**2,806**	**6.6**
2000	1,917	36.6	840	2.5	67	1.1	**2,824**	**6.4**
2001	2,301	43.1	1,538	4.4	63	1.0	**3,902**	**8.6**
2002	3,133	57.2	1,727	4.9	63	1.0	**4,923**	**10.7**
2003	2,695	47.8	2,091	5.9	55	0.9	**4,841**	**10.4**
2004	3,667	62.9	2,641	7.4	110	1.5	**6,418**	**13.5**
2005	3,516	58.3	2,885	7.8	108	1.7	**6,509**	**13.4**
2006	5,535	88.6	3,131	8.6	140	2.1	**8,806**	**18.0**
2007	4,832	75.0	2,991	8.1	163	2.4	**7,986**	**16.0**
2008	4,768	72.5	2,597	7.0	99	1.4	**7,464**	**14.8**
2009	10,233	154.4	2,488	6.7	147	2.1	**12,868**	**25.3**
2010	11,883	182.0	4,622	12.2	159	2.1	**16,664**	**32.2**
2011	16,467	247.7	5,697	14.9	237	3.1	**22,401**	**42.6**

* Coccidioidomycosis is endemic but not reportable in Texas.

**TABLE 2:** Number and incidence per 100,000 population of coccidioidomycosis cases, by region, age group, and sex — Arizona, California, Nevada, New Mexico, and Utah, 2011

Area and age group (yrs)	Male		Female		Total*	
		
	No.	Incidence	No.	Incidence	No.	Incidence
**Arizona**						
<5	38	16.5	25	11.3	**63**	**14.0**
5–19	621	89.4	796	120.3	**1,428**	**105.3**
20–39	1,671	187.1	2,707	319.4	**4,422**	**254.1**
40–59	2,239	276.4	3,223	386.5	**5,509**	**335.1**
60–79	1,919	382.4	2,078	372.8	**4,037**	**381.1**
≥80	424	445.4	461	334.8	**897**	**385.2**
All ages	6,954	215.7	9,349	286.9	**16,467**	**254.0**
**California**						
<5	35	2.7	22	1.8	**57**	**2.2**
5–19	340	8.5	263	6.9	**605**	**7.7**
20–39	1,329	24.0	561	10.6	**1,897**	**17.6**
40–59	1,513	30.0	583	11.4	**2,098**	**20.6**
60–79	509	21.4	337	12.4	**848**	**16.7**
≥80	88	18.5	75	9.8	**163**	**13.1**
All ages	3,839	20.5	1,844	9.7	**5,697**	**15.1**
**Nevada, New Mexico, and Utah**						
<5	0	0.0	0	0.0	**0**	**0.0**
5–19	8	0.9	5	0.6	**13**	**0.8**
20–39	15	1.4	17	1.7	**32**	**1.5**
40–59	49	5.2	29	3.0	**77**	**4.1**
60–79	62	12.2	38	7.2	**100**	**9.6**
≥80	10	11.6	4	3.2	**14**	**6.6**
All ages	143	3.8	93	2.5	**237**	**3.1**
**Total endemic area** †						
**<5**	**73**	**4.0**	**47**	**2.7**	**120**	**3.3**
**5–19**	**969**	**17.4**	**1,064**	**20.0**	**2,046**	**18.8**
**20–39**	**3,015**	**40.1**	**3,286**	**45.8**	**6,352**	**43.2**
**40–59**	**3,801**	**55.8**	**3,834**	**55.5**	**7,684**	**56.0**
**60–79**	**2,491**	**73.4**	**2,455**	**90.2**	**4,988**	**69.1**
**≥80**	**522**	**79.4**	**540**	**52.3**	**1,074**	**63.6**
**All ages**	**10,398**	**40.3**	**11,288**	**43.4**	**22,401**	**43.2**

* Categories might not sum to totals because of missing age and sex data.

† Coccidioidomycosis is endemic but not reportable in Texas.
